# The post‐ovulatory rise in progesterone is lower and the persistence of oestrous behaviour longer during the first compared with the second cycle of the breeding season in mares

**DOI:** 10.1111/rda.14273

**Published:** 2022-10-06

**Authors:** John R. Newcombe, Sandra Wilsher, Juan Cuervo‐Arango

**Affiliations:** ^1^ Warren House Farm Equine Fertility Clinic Brownhills UK; ^2^ Sharjah Equine Hospital Sharjah United Arab Emirates; ^3^ The Paul Mellon Laboratory of Equine Reproduction Newmarket UK; ^4^ Departamento de Medicina y Cirugía Animal, Facultad de Veterinaria Universidad Cardenal Herrera‐CEU, CEU Universities Valencia Spain

**Keywords:** first ovulation of the year, Mare, oestrous behaviour, progesterone, winter anoestrus

## Abstract

Mares are seasonally polyoestrous breeders. Therefore, the first ovulation of the season, following winter anoestrus, is the only cycle in which mares ovulate without the presence of an old CL from the previous cycle. The objective of this study was to compare the length of oestrous behaviour, and plasma progesterone concentrations during the early post‐ovulatory period between mares after the first and second ovulation of the breeding season. Overall, 38 mares and 167 oestrous periods were used in the study. From those, 11 mares were used during the first and subsequent oestrous period to measure and compare the post‐ovulatory rise in progesterone concentration, whereas all the mares were used to compare the length of the post‐ovulatory oestrous behaviour between the first and subsequent cycles of the breeding season. The persistence of the post‐ovulatory oestrus was longer (*p* < .001) following the first ovulation of the year (median of 52 h) compared with the subsequent ovulations (median of 36 h for second and later ovulations groups; *n* = 38 mares). The progesterone concentration at any of the four 8 h‐intervals analysed (28, 36, 76 and 84 h post‐ovulation) was lower (*p* < .01) following the first versus the second ovulation of the year. By 36 h post‐ovulation the progesterone concentration of mares at the second ovulation of the year had passed the threshold of 2 ng/ml (2.1 ± 0.33 ng/ml), whereas in the first cycle it was 1.2 ± 0.13 ng/ml. In conclusion, mares had lower progesterone concentrations in their peripheral circulation and longer persistence of oestrous behaviour following the first ovulation of the year compared with the second and subsequent ovulatory periods of the breeding season.

## 1 INTRODUCTION

The mare is a seasonally polyoestrous breeder characterized by cessation of cyclicity in the autumn as daylight decreases. Thus, most mares enter a period of anoestrus during winter (Aurich, 2011; Fitzgerald & McManus, 2000). The winter anoestrus is an anovulatory phase characterized by the absence of any major follicular activity and basal concentration of peripheral progesterone due to the absence of any active luteal tissues in the ovaries. The length of the anovulatory period is highly variable between mares, but in those that do enter anoestrus it lasts for at least 2 months (Fitzgerald & McManus, 2000). As the duration of natural ambient light increases in the spring cyclicity returns with the mean date of the first ovulation of the season in the Northern Hemisphere occurring around mid‐April (23 April, for this farm). However, inherent biological variation in an individual mare's response means that the first ovulation of the breeding season may occur anytime between March and May (Ginther, 1992).

Progesterone production by the corpus luteum (CL) begins relatively soon after ovulation with the first significant increase in plasma levels seen between 12 and 24 h post‐ovulation, before levels reach a plateau around Days 5 to 6 (Townson et al., 1989). Luteinizing hormone (LH), produced by the pituitary gland, is also believed to influence progesterone levels because of its luteotropic effect in the mare and possibly accounts for the early post‐ovulatory rise in progesterone concentrations (Ginther, 1992). On the other hand, despite seasonal changes in circulating LH (Aurich, 2011) no apparent differences in progesterone concentrations were observed between the mid‐ and late‐breeding seasons (Townson et al., 1989).

The oestrous or sexual behaviour of mares is a function of circulating follicular oestrogens and basal progesterone concentrations. Mares do not usually show signs of oestrus until peripheral progesterone concentrations have dropped to below 2 ng/ml, and oestrogen concentrations are beginning to rise (Nett et al., 1976). Oestrous behaviour usually ceases a day or two after ovulation (Ginther, 1992), at a time when progesterone has risen above the threshold of 2 ng/ml. This explains the long oestrus typically observed in mares preceding the first ovulation of the year when they are coming from winter anoestrus or spring transition and progesterone is basal. The leading author of this manuscript has observed over numerous years that post‐ovulatory oestrous behaviour persists longer following the first ovulation of the year compared with the subsequent cycles (Newcombe, personal observation). Whether this finding results from differences in the speed of post‐ovulatory rise of progesterone concentrations is unknown, and to date, has not been investigated.

Although the fertility of mares bred at the first cycle of the breeding season is reported to be similar to that observed in subsequent cycles (Cuervo‐Arango & Clark, 2010), a slower post‐ovulatory rise in progesterone following the first ovulation of the year may be a relevant factor to be considered in embryo transfer programs, in which the window of synchrony between recipients and embryos is influenced by the speed of progesterone rise post‐ovulation (Cuervo‐Arango et al., 2019a, 2019b).

The objective of this study was to compare the length of oestrous behaviour and plasma progesterone concentrations during the early post‐ovulatory period between mares in their first and second cycles of the breeding season. It was hypothesized that the oestrous behaviour would be longer, and progesterone concentrations lower in the first versus the second cycle.

## 2 MATERIALS AND METHODS

### 2.1 Animals

The resident mares at a fertility clinic in the UK (52° 37' N) were used in the study over several breeding seasons. Mares were aged 2 to 28 years and varied from pony to Shire horse but were mostly of riding horse type. Animals were fed hay and mineralized salts ad libitum as well as <1 kg of concentrate per day. Mares were kept in paddocks during the day and housed in boxes at night and starting from 1 December they were placed under artificial photoperiod for 16 h a day. Inclusion criteria were that mares had a period of winter anoestrus (i.e., absence of CL and no ovarian activity) >2 months before data collection at the 1st ovulation of the season and that post‐ovulatory teasing behaviour was available for at least the first two cycles (first and subsequent ovulations) of a given breeding season. Furthermore, only mares with single ovulations were included. In total, data from 38 mares and 167 oestrous cycles were used in the study.

### 2.2 Ultrasound examinations and teasing behaviour

Ultrasonography of the genital tract of mares was performed by the same experienced operator using the same ultrasound machine (Mindray DP‐10 vet equipped with a linear probe of 10 MHz). Starting in January, anoestrous mares were monitored every 7 to 10 days for the follicular activity. Once a follicle reached 30 mm, the frequency of ultrasound examinations increased to every 2 to 3 days, and as the follicle reached the peri‐ovulatory stage examinations were undertaken thrice daily (every 8 h) to detect ovulation. Once ovulation had occurred, mares were presented every 8 h to the same teasing pony stallion to determine the presence or absence of oestrous behaviour. The length of post‐ovulatory oestrous behaviour was calculated from the first post‐ovulatory examination until the last interval of full oestrus (Grade 2).

Oestrous behaviour after ovulation was graded as follows:

#### 2.2.1 Grade 2 (full oestrus)

The mare approached the stallion willingly and was allowed to make nose‐to‐nose contact over a low barrier. On initial contact, or after swinging her hind quarters towards the stallion, the mare widened the stance of her hind legs, flexed the hocks and with tail raised, everted the clitoris repeatedly and passed either a single large or multiple small amount(s) of urine.

#### 2.2.2 Grade 1

Behaviour similar to Grade 2 but less positive and some initial negative signs such as squealing. The position of urination was not adopted. A more positive attitude towards the stallion was maintained with continuous eversion of the clitoris, giving the impression that she would not resist mating.

#### 2.2.3 Grade 0

Mares which were not in oestrus exhibited some, but not necessarily all, of the following behavioural characteristics; reluctance to approach the stallion; desire to swing hindquarter towards the stallion and at least threaten to kick or even kick out viciously; tail held down tightly or swishing “angrily” from side to side; ears held back; threatening to bite; striking out with front legs; squealing. Overall, giving a definite impression that she would resist mating.

A total of 167 oestrous cycles were evaluated for post‐ovulatory oestrous behaviour (38 from first ovulation, 38 from the subsequent second ovulation in the same season, and 91 from 3rd, 4th, or more cycles over different seasons).

### 2.3 Blood samples and progesterone determination

A subset of 11 mares were used to obtain progesterone data. Jugular heparinized blood samples were taken at 28, 36, 76 and 84 h post‐ovulation during the first and second cycles of the 2019 breeding season. Blood samples were centrifuged at 2000 G and aliquots of plasma were frozen and stored for later assay determination.

Progesterone concentrations in plasma samples were measured in duplicate using a commercial ELISA assay (DRG Instruments, Marburg, Germany). Dilution curves generated from a pool of equine plasma with a known high concentration of progesterone and the 0 ng/ml standard supplied with the kit, recommended for serial dilution by the manufacturer, were parallel to the curve produced with the standards supplied with the kit. The intra‐ and inter‐assay coefficient variations (CVs) were 6.4% and 6.6%, respectively. The minimal detectable concentration of the assay was 0.16 ng/ml, calculated by adding two standard deviations to the mean optical density value of 10 zero standard replicates and determining the corresponding concentration of progesterone from the standard curve. Cross reactivities were reported as 0.35% for Pregnenolone, 0.3% for 17alpha OH Progesterone, 1.1% for 11‐Desoxycorticosterone and 0.2% for Corticosterone. Cross‐reactivity to equine 5alpha‐pregnanes was not determined.

### 2.4 Statistical analyses

All the data were computed using the statistical software Systat 13 (Inpixon HQ: Palo Alto, CA, USA). Progesterone concentration data were analysed by a general linear model of variance with a repeated statement to account for autocorrelation between sequential observations. Within each 8 h intervals, progesterone data were examined further by a paired Student's *t*‐test. The effect of oestrous cycle order within the breeding season (1st, 2nd and 3rd or further) on the length of post‐ovulatory oestrous behaviour was determined by the non‐parametric Kruskal–Wallis test. A probability of *p* ≤ .05 indicated that a difference was significant.

## 3 RESULTS

The first ovulation of the year following the winter anoestrus of the 11 mares in which progesterone concentrations were determined took place between 1 February and 7 May 2019. On average, the subsequent second ovulation of the year in the same mares occurred 25.3 ± 1.8 days (20 to 29 days) later.

**FIGURE 1:**
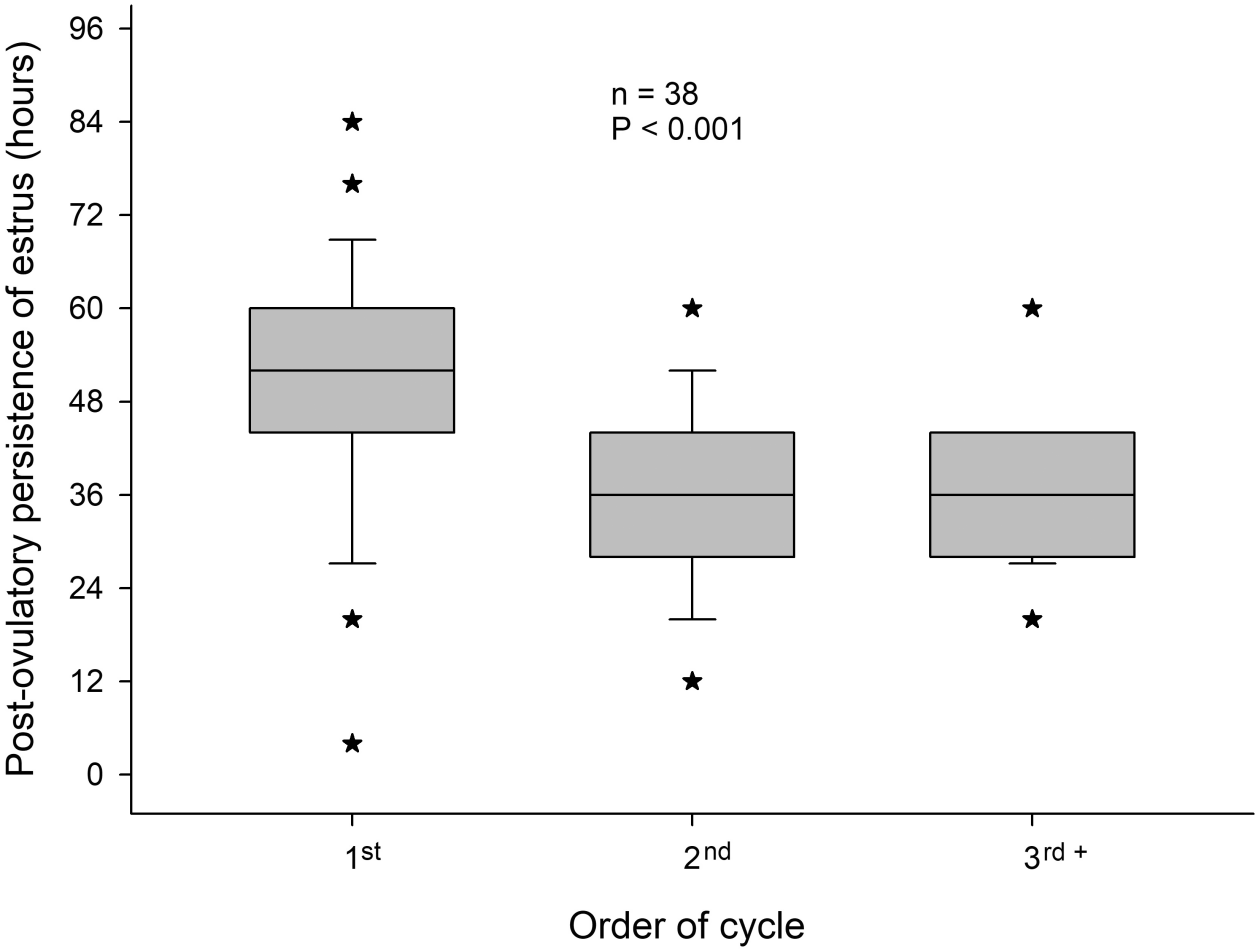
Box plot of oestrous length for the post‐ovulatory period of the 1st, 2nd and 3rd or subsequent cycles of 38 mares. The asterisks indicate outliers. The median length for the first, second or subsequent cycles are 52, 36, and 36 h, respectively. The post‐ovulatory persistence of oestrus was longer in the first cycle (*p* < .001) than in the subsequent cycles.

The median post‐ovulatory length of oestrous behaviour for the 167 oestrous cycles evaluated in the study was 44 h (0 to 84 h). The mean persistence of oestrus was longer (*p* < .001) following the first ovulation of the year (52 h) versus subsequent ovulations (median of 36 h for both groups; Figure 1). From the 38 mares, only one mare (a 2‐year‐old maiden) did not show oestrus at all (Grade 0) at the first cycle of the year, neither before nor after ovulation. During the subsequent oestrous periods, she showed more positive oestrous behaviour.

**FIGURE 2:**
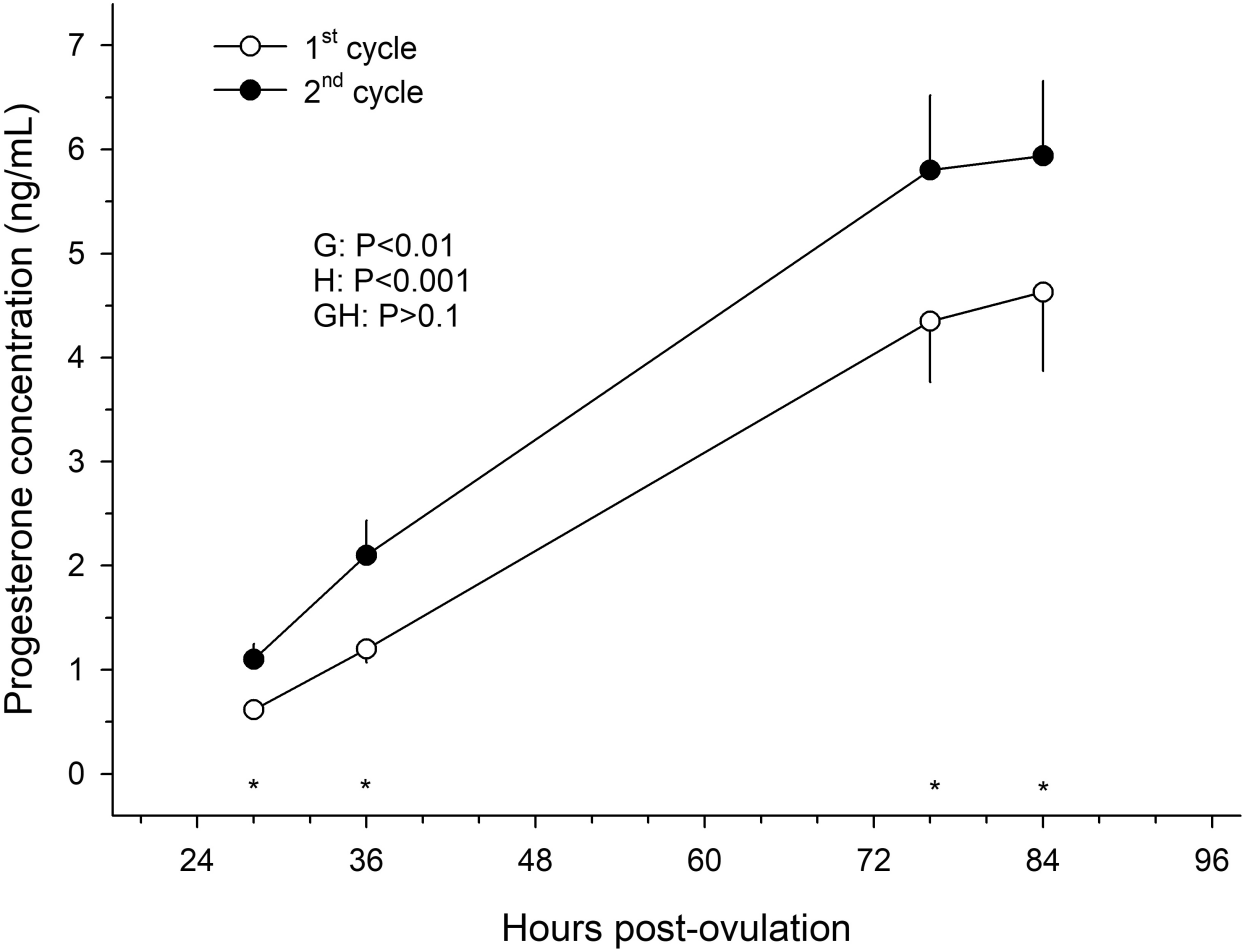
Mean (± S.E.M.) progesterone concentration from 11 mares during the first and second early post‐ovulatory periods of the breeding season. Probabilities for the main effects of group (G) and hour (H) and the group‐by‐hour interaction (GH) are shown. Asterisks (*) denote a difference (*p* < .05) in progesterone concentration between groups (1st vs. 2nd ovulations) at a given time after ovulation.

The progesterone concentration at any of the four time periods analysed was significantly lower *(p <* .01) following the first ovulation of the year than during the subsequent cycle (Figure 2). By 36 h post‐ovulation, the progesterone concentration of mares at the second ovulation of the year had passed the threshold of 2 ng/ml (2.1 ± 0.33 ng/ml), whereas in the first cycle it was 1.2 ± 0.13 ng/ml.

To investigate the effect of month on progesterone production by the early CL following the first ovulation of the year, progesterone concentration at 28 h post‐ovulation was compared between mares ovulating for the first time in February (*n* = 5) and those ovulating in April (*n* = 3) and May (*n* = 1). The mean ± S.E.M. progesterone concentration 28 h post‐ovulation for mares ovulating in February was 0.68 ± 0.11 ng/ml, which was not different (*p* > .05) from that of mares ovulating in April–May (0.57 ± 0.13 ng/ml).

## 4 DISCUSSION

The hypothesis that progesterone concentrations would be lower during the early post‐ovulatory period of the first ovulation of the year compared with the subsequent cycle is substantiated by the results of this study. One confounding factor which might account for this difference would be that the first ovulation of the year occurs on average 3 to 4 weeks earlier (equivalent to an oestrous cycle length) in the season than the second ovulation. One possible explanation for this could be the seasonal variations in LH levels in the circulation (Aurich, 2011), which are lower in February than in April (Ginther, 1992). However, despite the luteotrophic effects of LH in promoting progesterone production by the CL, a close analysis of the effect of month on progesterone concentration in mares following the first ovulation of the year showed no significant difference. Hence, mares ovulating in February for the first time had similar progesterone concentrations to those ovulating in April and May. This concurs with a previous study showing that progesterone concentrations in cycling mares did not vary significantly between the mid‐ and late‐breeding seasons (Townson et al., 1989). A plausible reason that could account for the observed difference in progesterone concentration between the first and subsequent ovulations may be the lack of the presence of a regressed CL or corpus albicans in the former compared to the latter. Since mares are seasonally polyoestrous breeders, the first ovulation of the season following winter anoestrus, is the only cycle in which mares ovulate without the presence of old luteal tissue in their ovaries from a previous cycle. Perhaps, persisting luteal tissue from the first cycle may contribute to the progesterone production alongside that of the newly formed CL during the second and subsequent cycles of the breeding season. Bergfelt et al. (2006) showed the ability of the CL to undergo resurgence following exogenous‐luteolysis by PGF2α. Comparatively, the functional resurgence is also accompanied by structural resurgence during early pregnancy in mares (Bergfelt & Ginther, 1989, 1996). Other studies have shown the ability of the luteal cells from both primary and secondary CLs in pregnancy to respond to the LH component of equine Chorionic Gonadotrophin (eCG) by secreting both oestrogens and progesterone (Daels et al., 1998; Sirois et al., 1990). Hence, luteal cells, albeit ones that have not been quelled in their production of progesterone by prostaglandin, can be “rescued” and stimulated to produce steroid hormones for an extended time period.

An early study (Evans et al., 1975) went further and cultured tissue from old corpora lutea from previous cycles (21 to 32 days old corpora albicans) as well as the CL (1 to 14 days old) from current cycles to compare the ability to synthesis progesterone in vitro. The latter study concluded that although the retention of conversion activity by the CL of the previous oestrous cycle was variable, the single CL of one mare from the previous ovulation (21 days old) had greater progesterone synthesising activity than the two more recent CL which were 2 and 3 days old at the time of ovariectomy (Evans et al., 1975).

The hypothesis that the persistence of post‐ovulatory oestrus would be longer during the first ovulation of the year is also substantiated by the results of this study. The fact that the length of post‐ovulatory oestrous behaviour does not shorten as the season advances (the 3rd and further cycles had similar lengths to that of the second cycle), points towards a regulatory role for progesterone rather than photoperiod on the length of oestrus. The threshold of progesterone concentrations in peripheral blood upon which oestrous behaviour ends has been set at 2 ng/ml (Nett et al., 1976). This progesterone threshold occurred around 36 h after ovulation in mares during the second cycle of the year, which coincided with the length of post‐ovulatory oestrous behaviour in the same group of mares. A delay of 16 h in the persistence of oestrus was observed in mares after the first ovulation of the year. Indirectly, it can be assumed, therefore, that the delay in the postovulatory rise in plasma progesterone concentration during the first ovulation of the year is equivalent to 16 h.

## 5 CONCLUSIONS

The results of this study showed that the first ovulation of the year in mares after a period of winter anoestrus of more than 2 months results in a persistence of post‐ovulatory oestrous behaviour equivalent to 16 h longer. In addition, the first ovulation of the season is characterized by initial lower progesterone production compared with the subsequent oestrous cycles.

## AUTHOR CONTRIBUTIONS

JRN conceived the study and collected the clinical data. SW performed progesterone assay and JCA designed the experiment, performed statistical analyses and wrote the manuscript. All the authors edited, revised and accepted the manuscript.

## CONFLICT OF INTEREST

None of the authors have any conflict of interest to declare.

## Data Availability

The data is available from the corresponding author upon request.
